# Supplementary material for: Chilensosides E, F, and G—New Tetrasulfated Triterpene Glycosides from the Sea Cucumber Paracaudina chilensis (Caudinidae, Molpadida): Structures, Activity, and Biogenesis
Source: Mar Drugs. 2023 Feb 5;21(2):114. doi: 10.3390/md21020114 (PMC9964569; doi:10.3390/md21020114)
Supplement: Supplementary file 1 [file marinedrugs-21-00114-s001.zip › marinedrugs-2185008-supplementary.pdf]

Supplementary data content page

Title: Chilensosides E, F, and G – New Tetrasulfated Triterpene Glycosides from the Sea Cucumber *Paracaudina chilensis* (Caudinidae, Molpadida): Structures, Activity and Biogenesis.

Authors: Alexandra S. Silchenko, Sergey A. Avilov, Roman S. Popov, Pavel S. Dmitrenok, Ekaterina A. Chingizova, Boris B. Grebnev, Anton B. Rasin and Vladimir I. Kalinin*

Address: *G.B. Elyakov Pacific Institute of Bioorganic Chemistry, Far Eastern Branch of Russian Academy of Sciences, Pr. 100-let Vladivostoka 159, 690022 Vladivostok, Russia*

Correspondence: kalininv@piboc.dvo.ru; Tel.: +7-423-231-1168

Content:

Figure S1. The ^{13}C NMR (125.67 MHz) spectrum of chilensoside E (**1**) in C_5D_5N/D_2O (4/1)

Figure S2. The ^{1}H NMR (500.12 MHz) spectrum of chilensoside E (**1**) in C_5D_5N/D_2O (4/1)

Figure S3. The COSY (500.12 MHz) spectrum of chilensoside E (**1**) in C_5D_5N/D_2O (4/1)

Figure S4. The HSQC (500.12 MHz) spectrum of chilensoside E (**1**) in C_5D_5N/D_2O (4/1)

Figure S5. The ROESY (500.12 MHz) spectrum of chilensoside E (**1**) in C_5D_5N/D_2O (4/1)

Figure S6. The HMBC (500.12 MHz) spectrum of chilensoside E (**1**) in C_5D_5N/D_2O (4/1)

Figure S7. 1 D TOCSY (500.12 MHz) spectra of Xyl1, Qui2, Glc3, Glc4, MeGlc5 of chilensoside E (**1**) in C_5D_5N/D_2O (4/1)

Figure S8. HR-ESI-MS and ESI-MS/MS spectra of chilensoside E (**1**)

Figure S9. The ^{13}C NMR (125.67 MHz) spectrum of chilensoside F (**2**) in C_5D_5N/D_2O (4/1)

Figure S10. The ^{1}H NMR (500.12 MHz) spectrum of chilensoside F (**2**) in C_5D_5N/D_2O (4/1)

Figure S11. The COSY (500.12 MHz) spectrum of chilensoside F (**2**) in C_5D_5N/D_2O (4/1)

Figure S12. The HSQC (500.12 MHz) spectrum of chilensoside F (**2**) in C_5D_5N/D_2O (4/1)

Figure S13. The HMBC (500.12 MHz) spectrum of chilensoside F (**2**) in C_5D_5N/D_2O (4/1)

Figure S14. The ROESY (500.12 MHz) spectrum of chilensoside F (**2**) in C_5D_5N/D_2O (4/1)

Figure S15. 1D TOCSY (500.12 MHz) spectra of Xyl1, Qui2, Glc3, Glc4, MeGlc5 of chilensoside F (**2**) in C_5D_5N/D_2O (4/1)

Figure S16. HR-ESI-MS and ESI-MS/MS spectra of chilensoside F (**2**)

Table S1. ^{13}C and ^{1}H NMR chemical shifts, HMBC and ROESY correlations of the aglycone part of chilensoside F (**2**)

Figure S17. The ^{13}C NMR (125.67 MHz) spectrum of chilensoside G (**3**) in C_5D_5N/D_2O (4/1)

Figure S18. The ^{1}H NMR (500.12 MHz) spectrum of chilensoside G (**3**) in C_5D_5N/D_2O (4/1)

Figure S19. The COSY (500.12 MHz) spectrum of chilensoside G (**3**) in C_5D_5N/D_2O (4/1)

Figure S20. The HSQC (500.12 MHz) spectrum of chilensoside G (**3**) in C_5D_5N/D_2O (4/1)

Figure S21. The ROESY (500.12 MHz) spectrum of chilensoside G (**3**) in C_5D_5N/D_2O (4/1)

Figure S22. The HMBC (500.12 MHz) spectrum of chilensoside G (**3**) in C_5D_5N/D_2O (4/1)

Figure S23. 1D TOCSY (500.12 MHz) spectra of Xyl1, Qui2, Glc3, Glc4, Glc5, MeGlc6 of chilensoside G (**3**) in C_5D_5N/D_2O (4/1)

Figure S24. HR-ESI-MS and ESI-MS/MS spectra of chilensoside G (**3**)

Table S2. ^{13}C and ^{1}H NMR chemical shifts, HMBC and ROESY correlations of the aglycone part of chilensoside G (**3**)

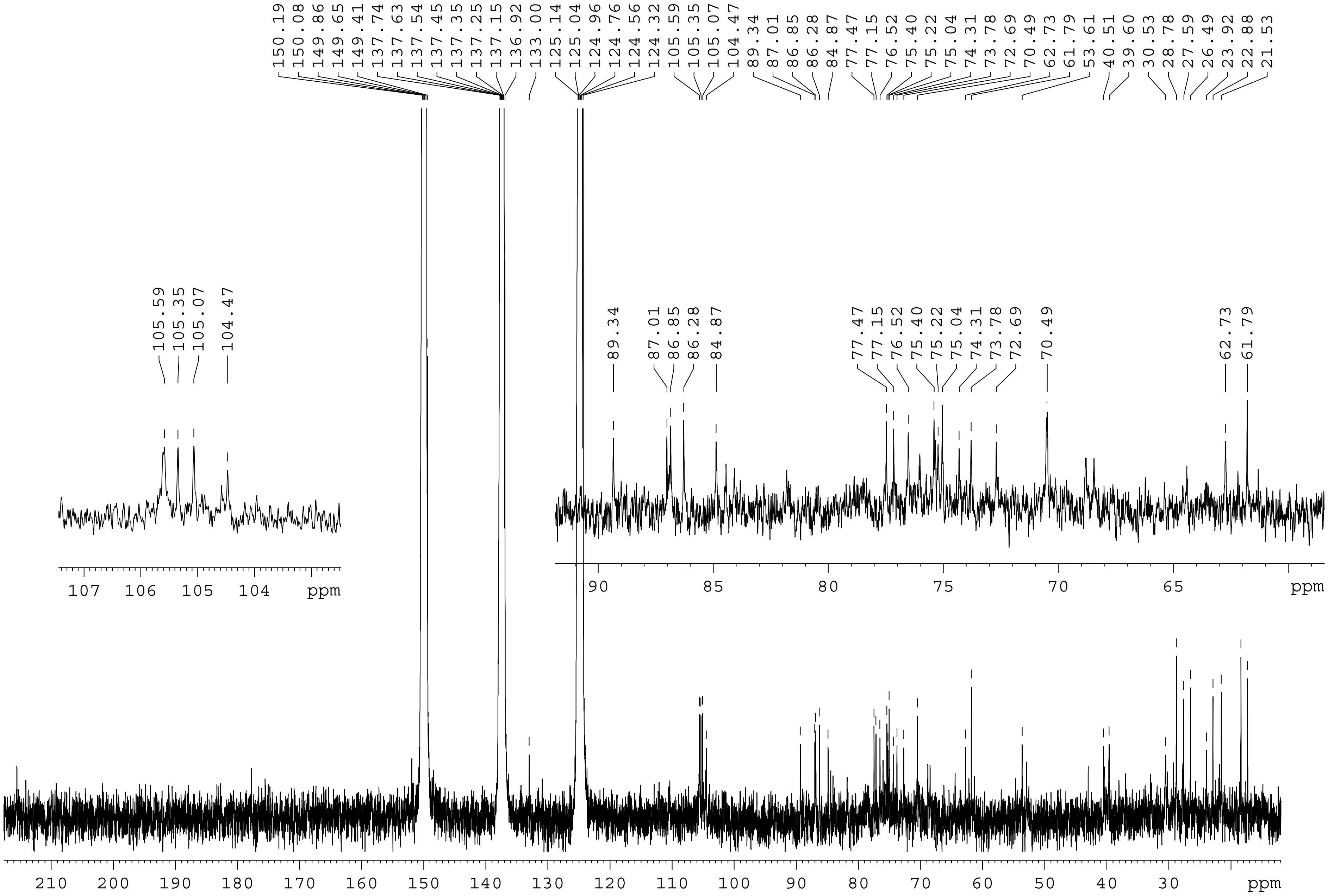

Figure S1. The ^{13}C NMR (125.67 MHz) spectrum of chilensoside E (**1**) in C_5D_5N/D_2O (4/1)

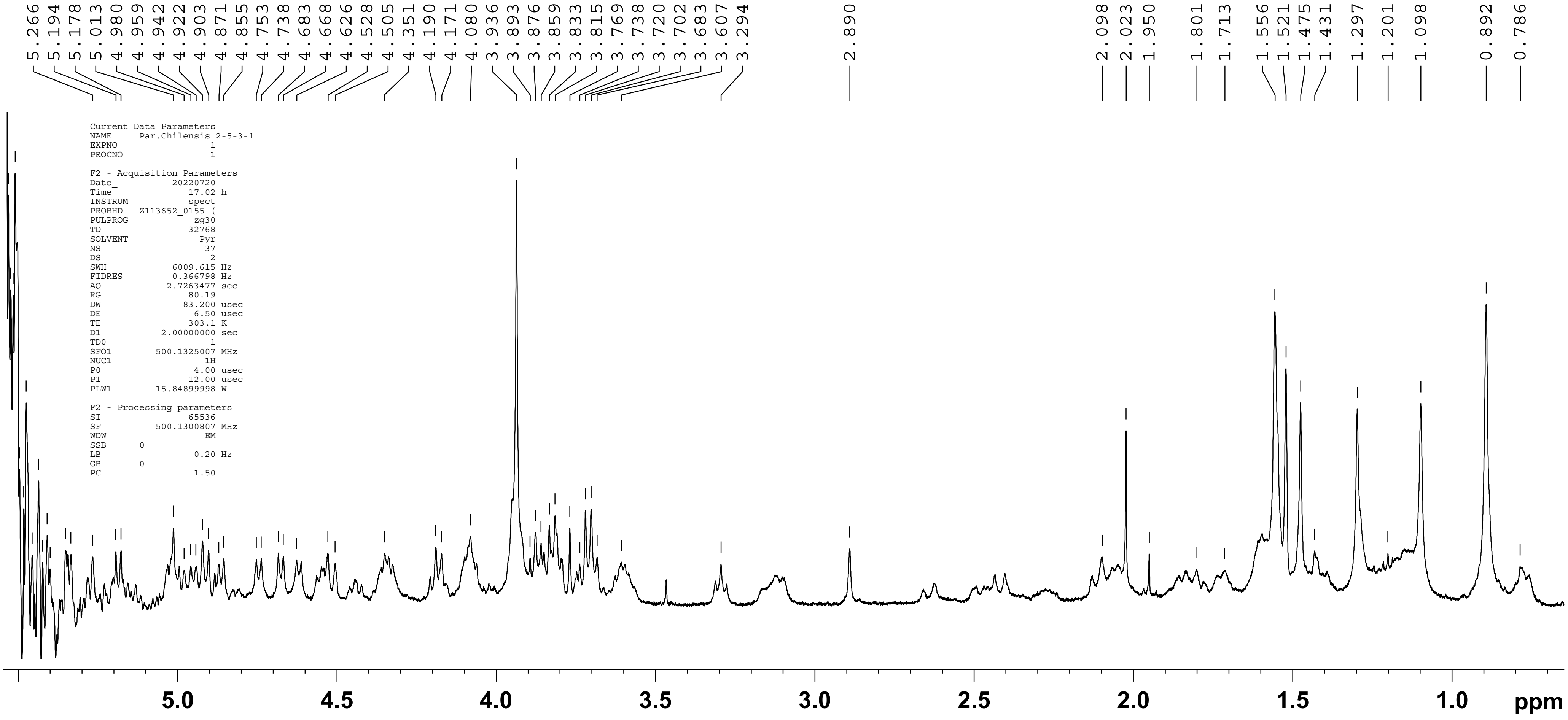

Figure S2. The ^{1}H NMR (500.12 MHz) spectrum of chilensoside E (**1**) in C_5D_5N/D_2O (4/1)

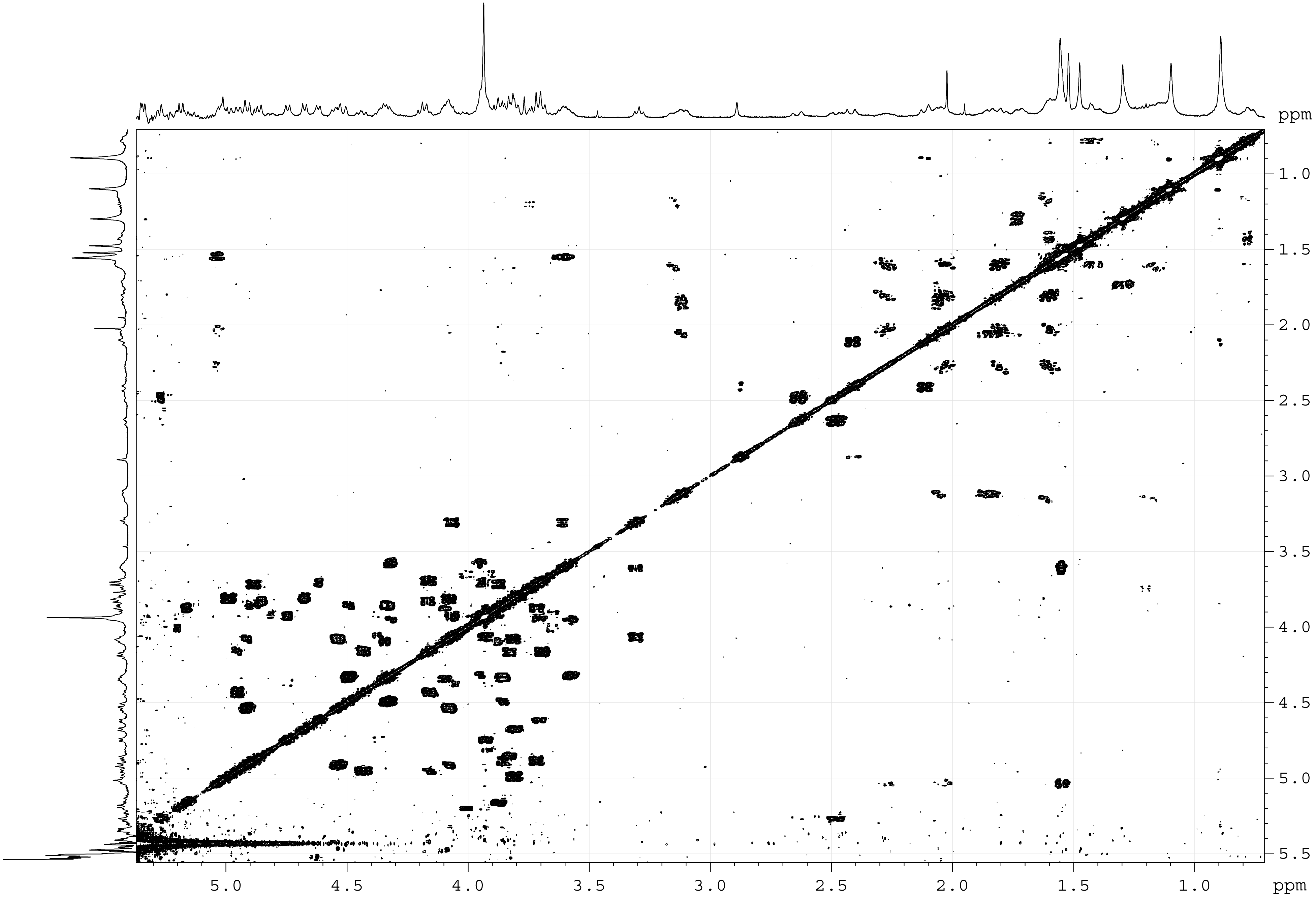

Figure S3. The COSY (500.12 MHz) spectrum of chilensoside E (**1**) in C_5D_5N/D_2O (4/1)

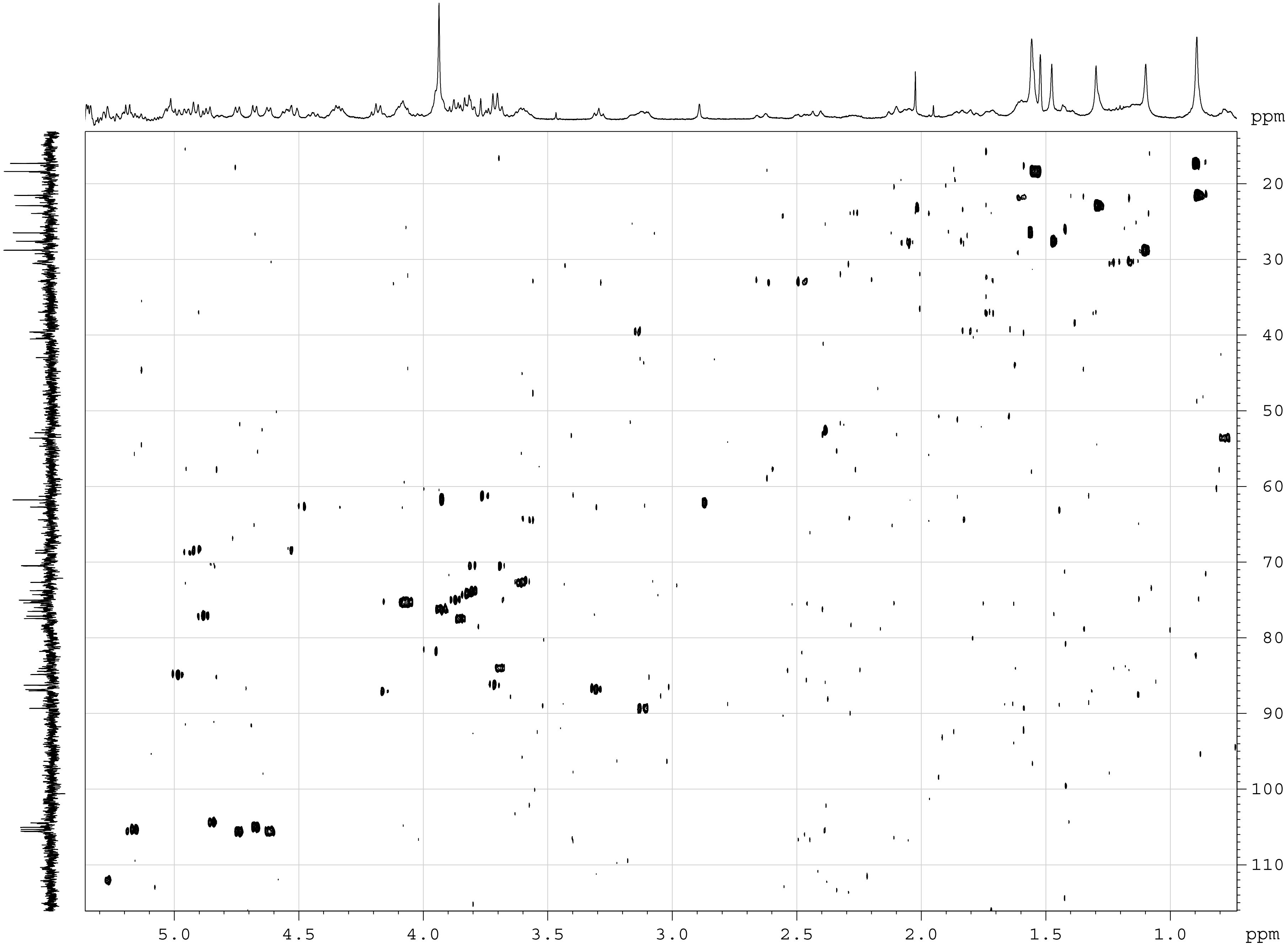

Figure S4. The HSQC (500.12 MHz) spectrum of chilensoside E (**1**) in C_5D_5N/D_2O (4/1)

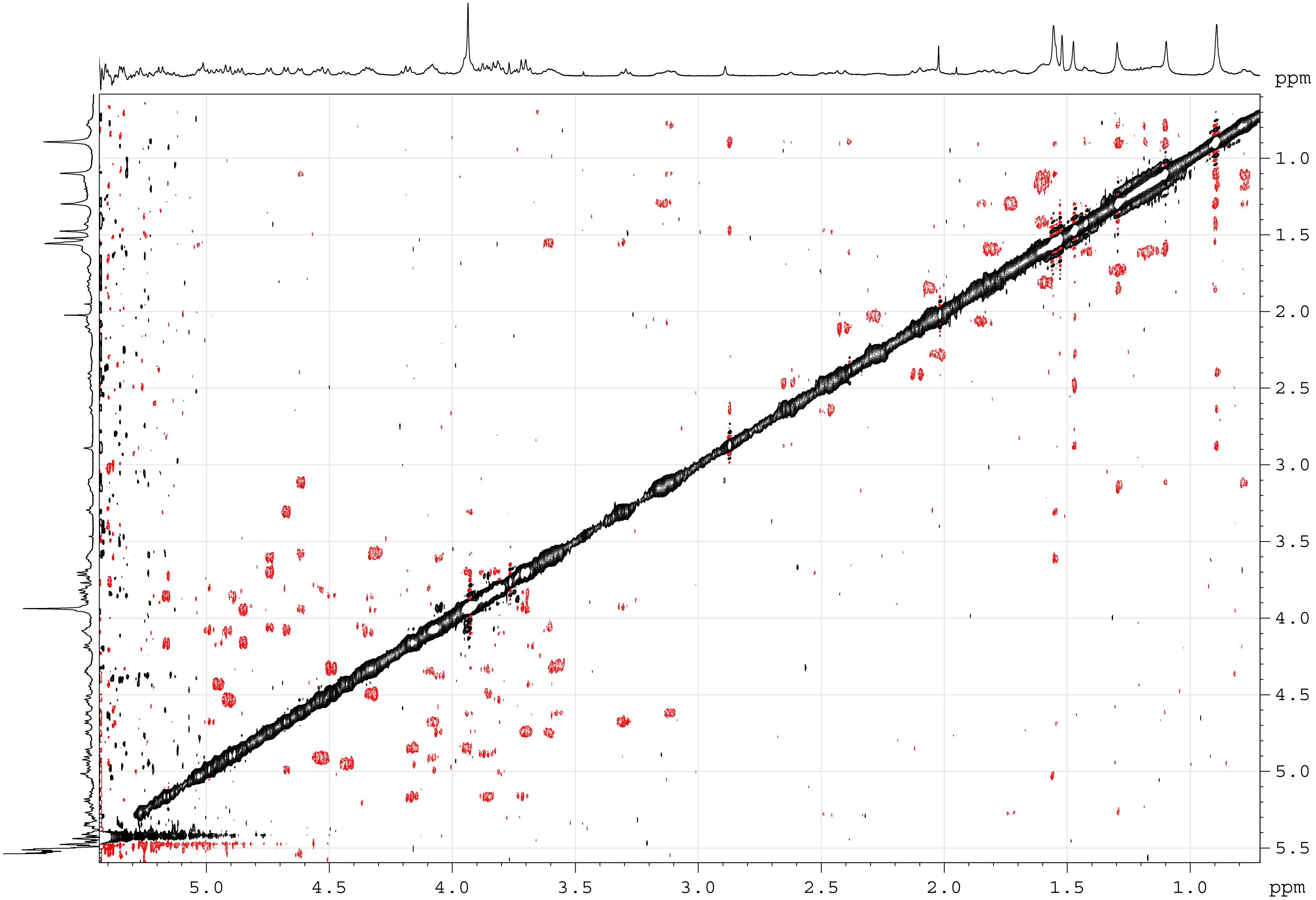

Figure S5. The ROESY (500.12 MHz) spectrum of chilensoside E (**1**) in C_5D_5N/D_2O (4/1)

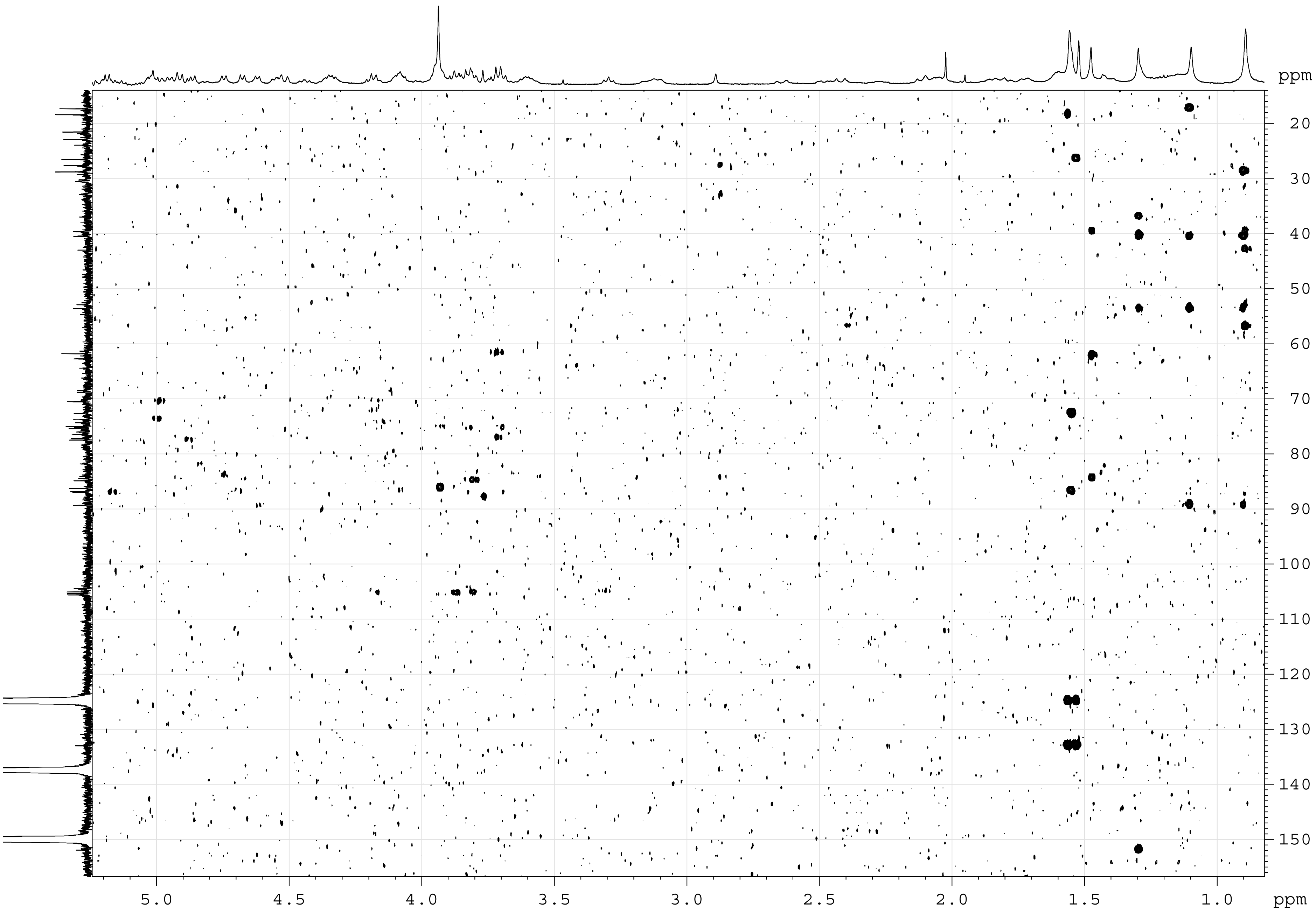

Figure S6. The HMBC (500.12 MHz) spectrum of chilensoside E (**1**) in C_5D_5N/D_2O (4/1)

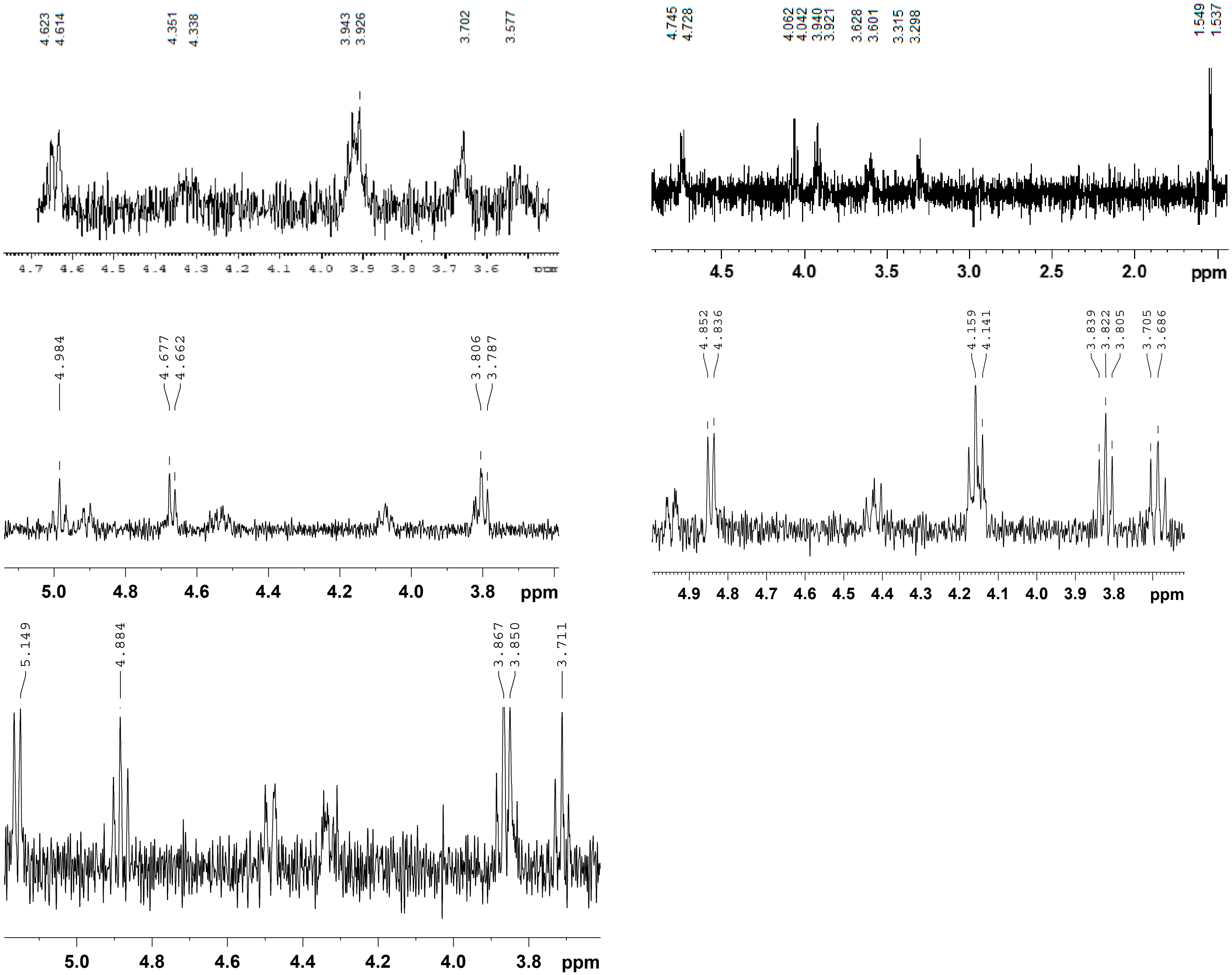

Figure S7. 1 D TOCSY (700.13 MHz) spectra of Xyl1, Qui2, Glc3, Glc4, MeGlc5 of chilensoside E (**1**) in C_5D_5N/D_2O (4/1)

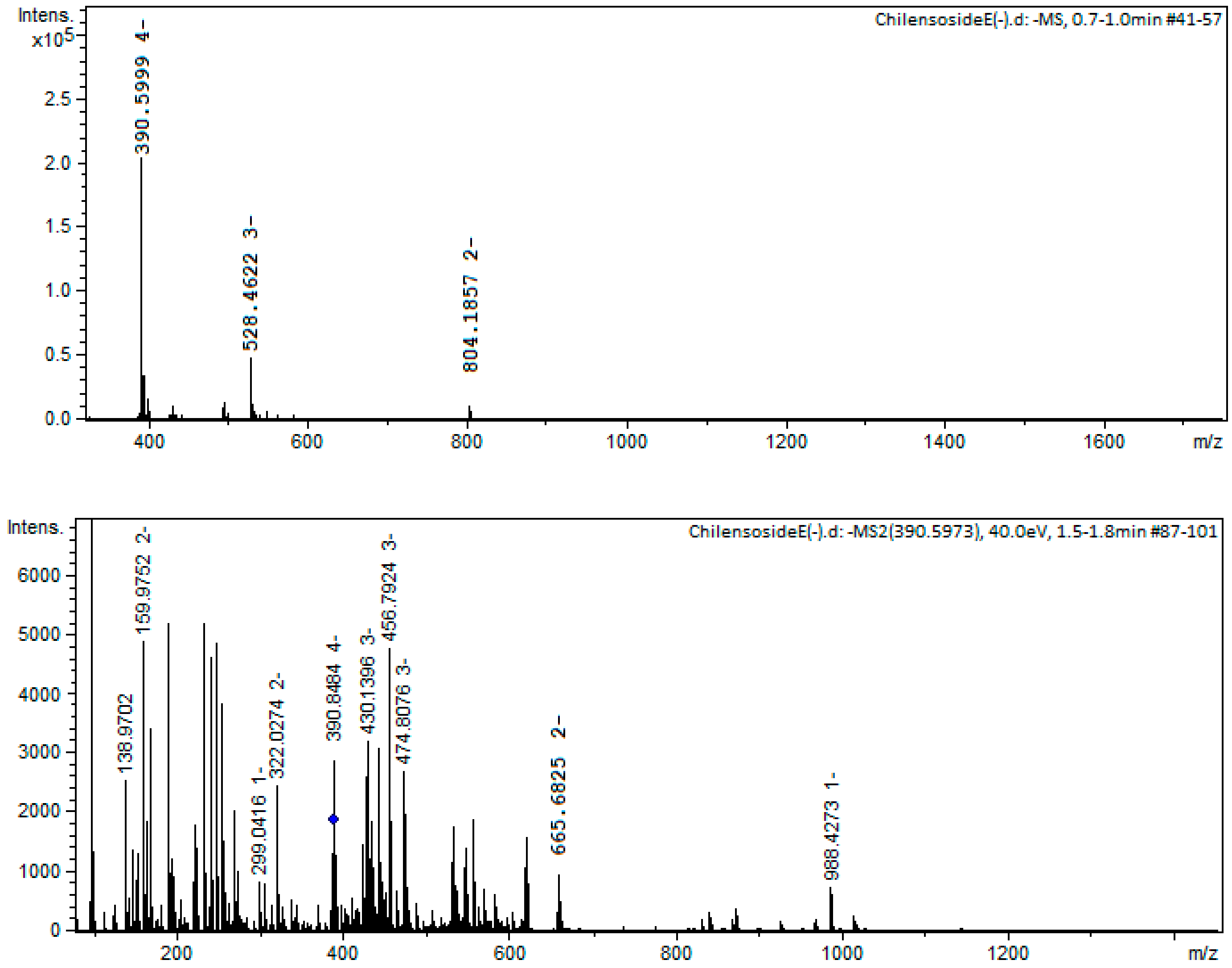

Figure S8. HR-ESI-MS and ESI-MS/MS spectra of chilensoside E (**1**)

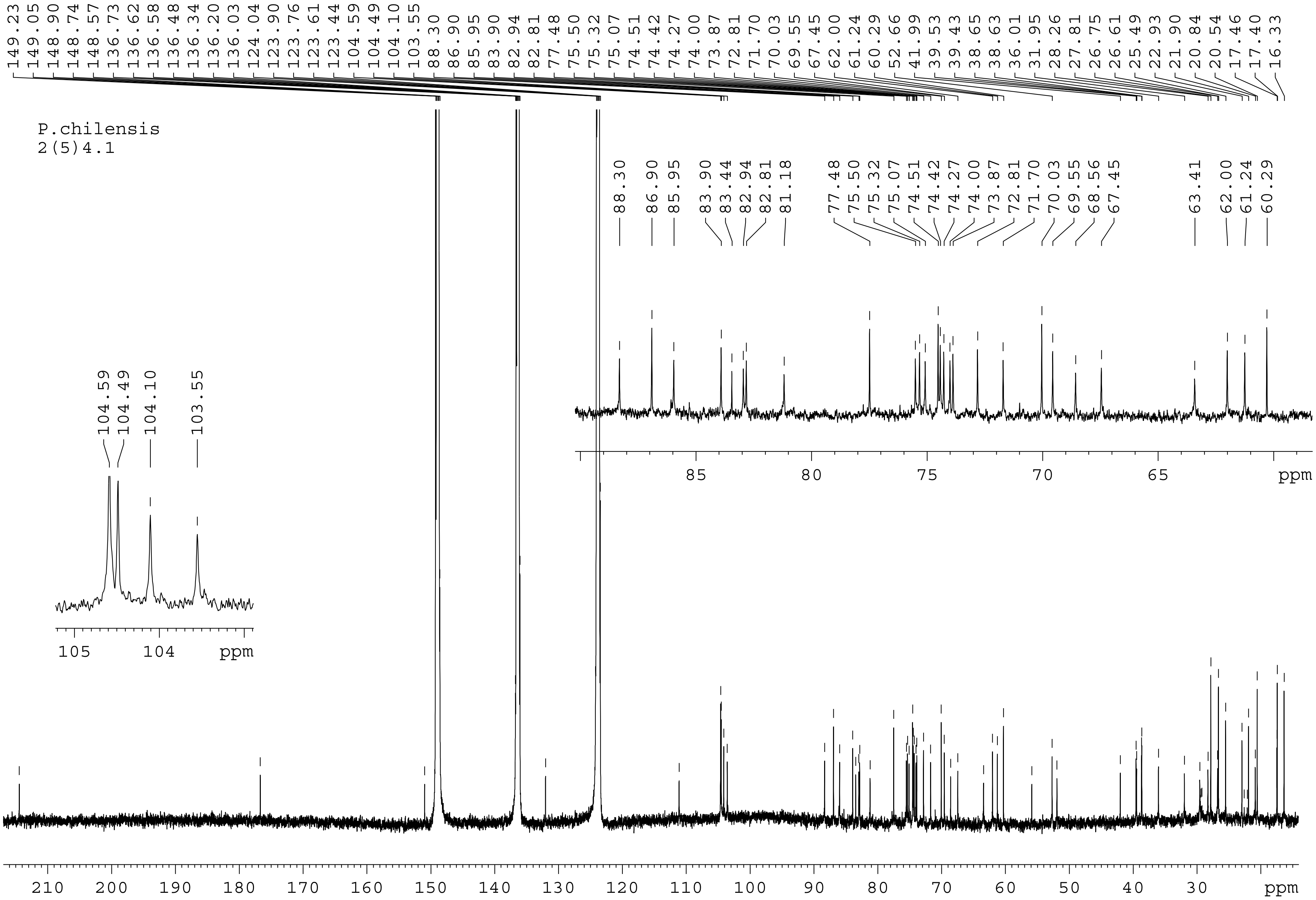

Figure S9. The ^{13}C NMR (125.67 MHz) spectrum of chilensoside F (**2**) in C_5D_5N/D_2O (4/1)

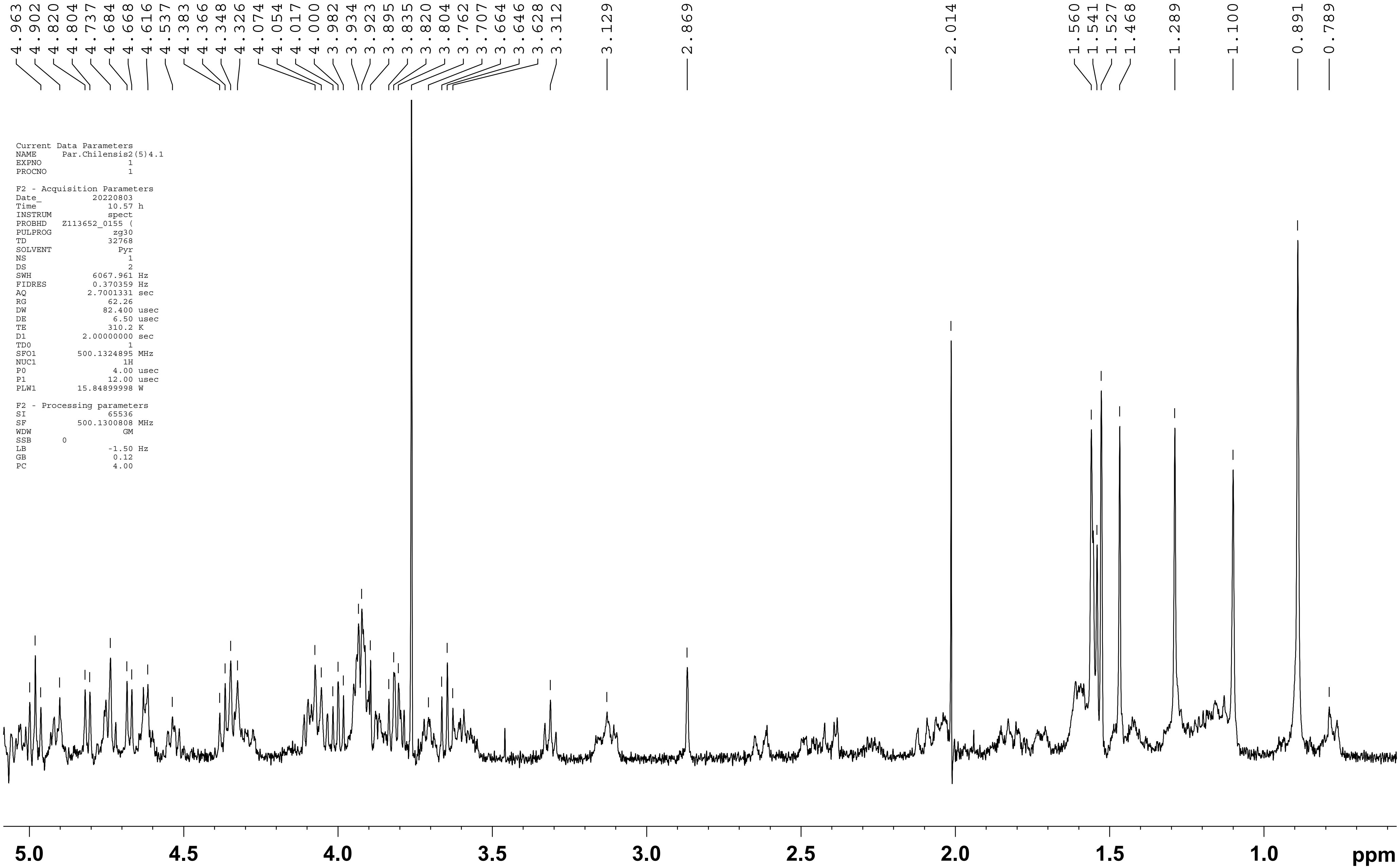

Figure S10. The ^{1}H NMR (500.12 MHz) spectrum of chilensoside F (**2**) in C_5D_5N/D_2O (4/1)

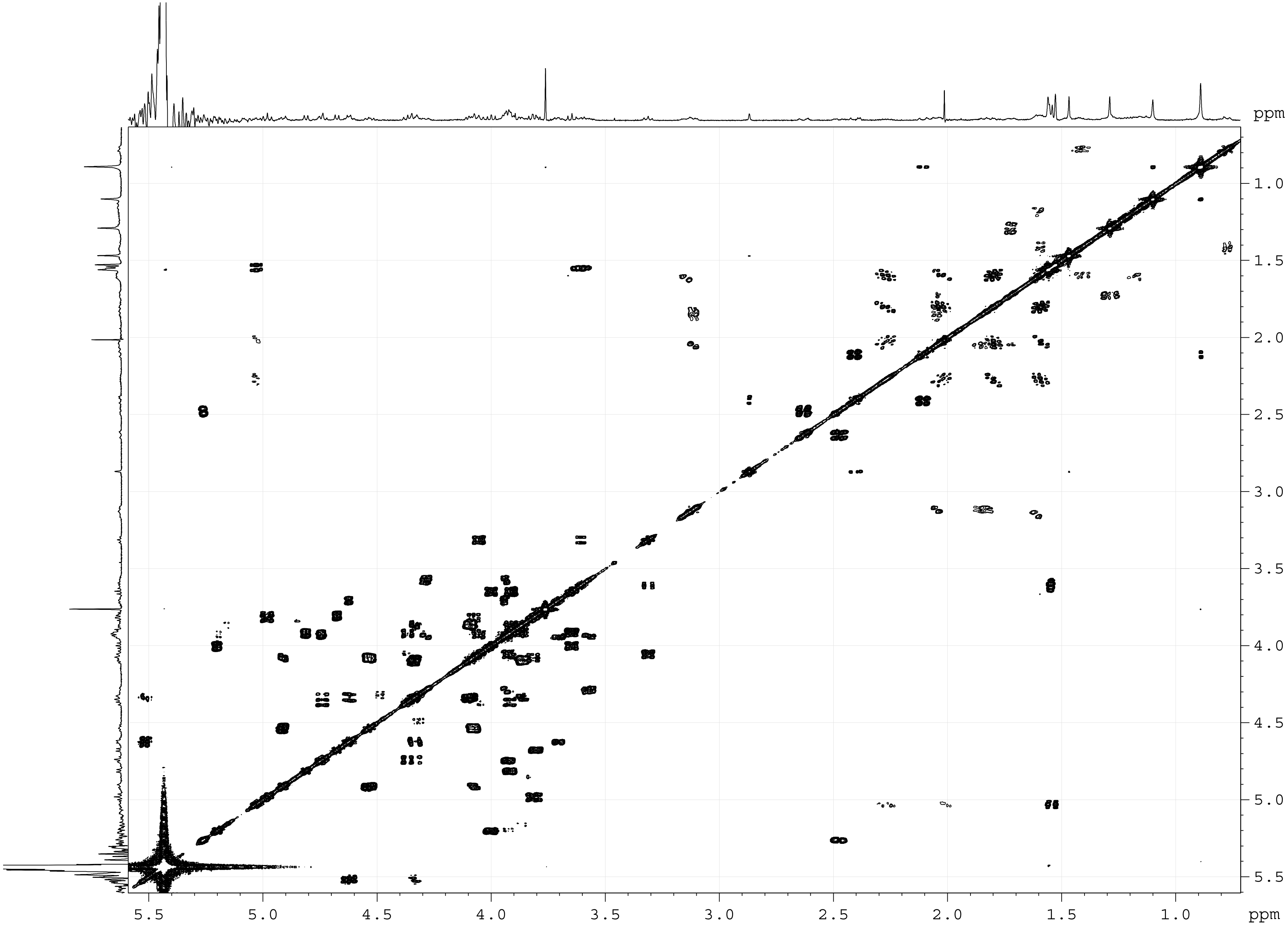

Figure S11. The COSY (500.12 MHz) spectrum of chilensoside F (**2**) in C_5D_5N/D_2O (4/1)

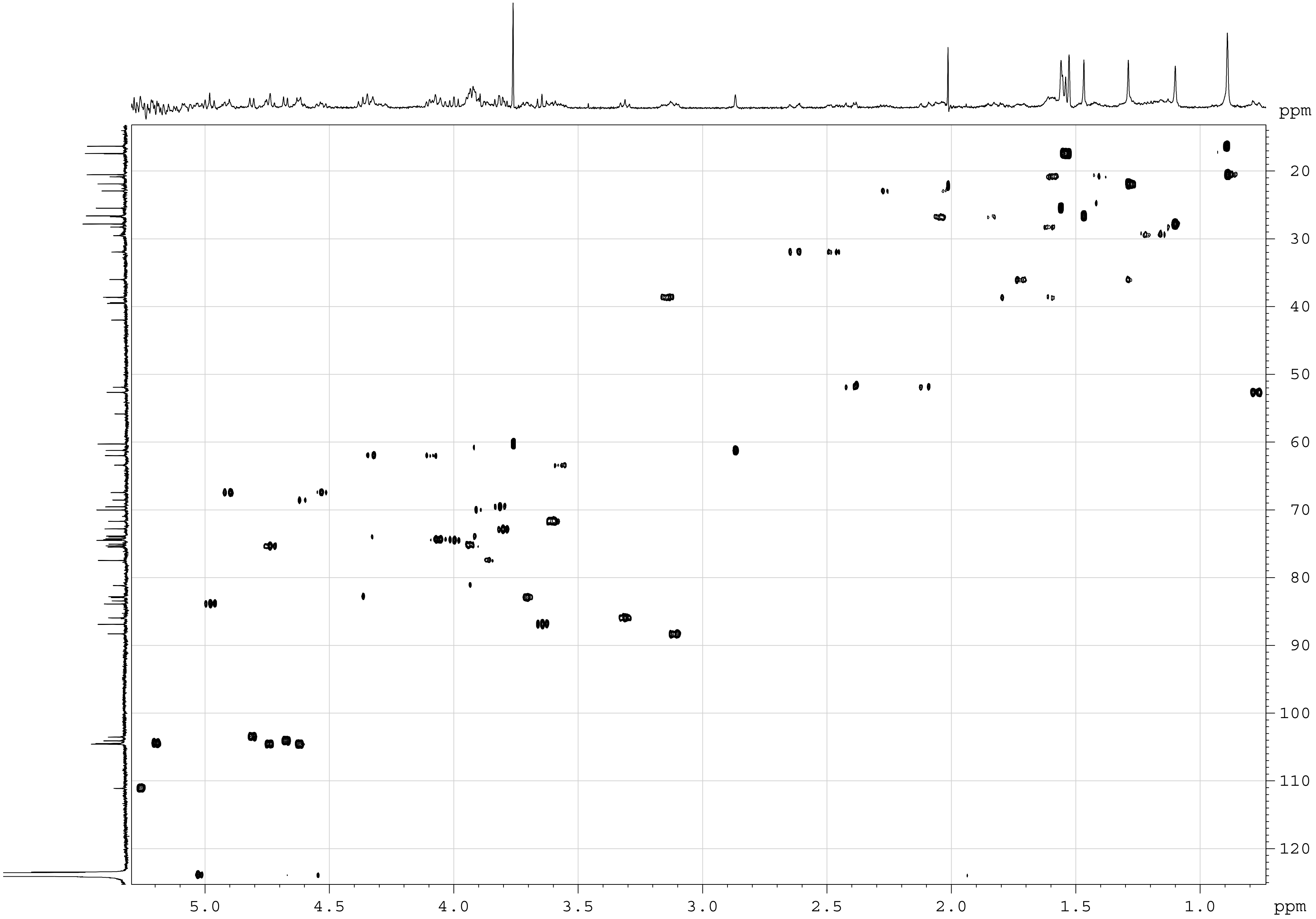

Figure S12. The HSQC (500.12 MHz) spectrum of chilensoside F (**2**) in C_5D_5N/D_2O (4/1)

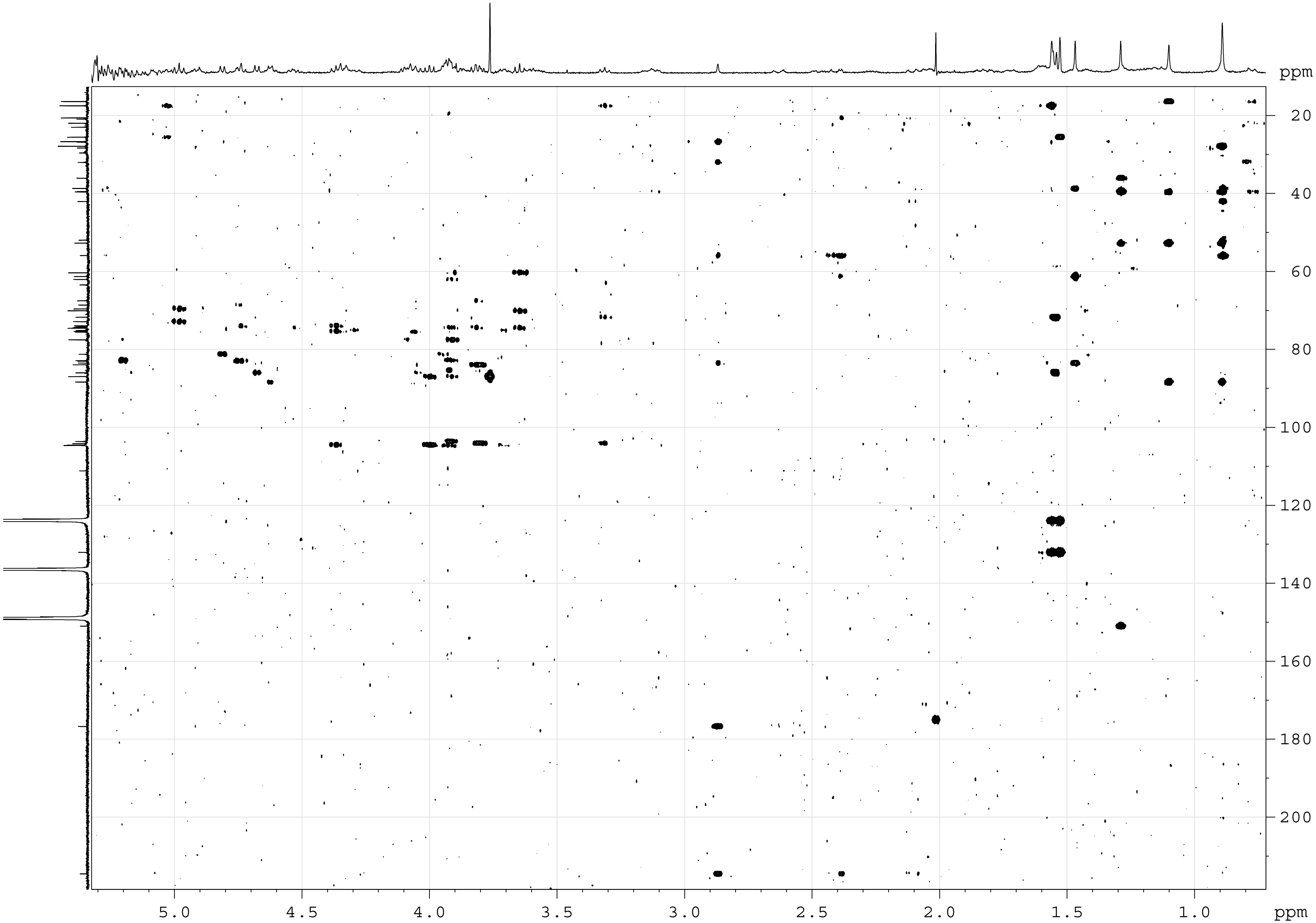

Figure S13. The HMBC (500.12 MHz) spectrum of chilensoside F (**2**) in C_5D_5N/D_2O (4/1)

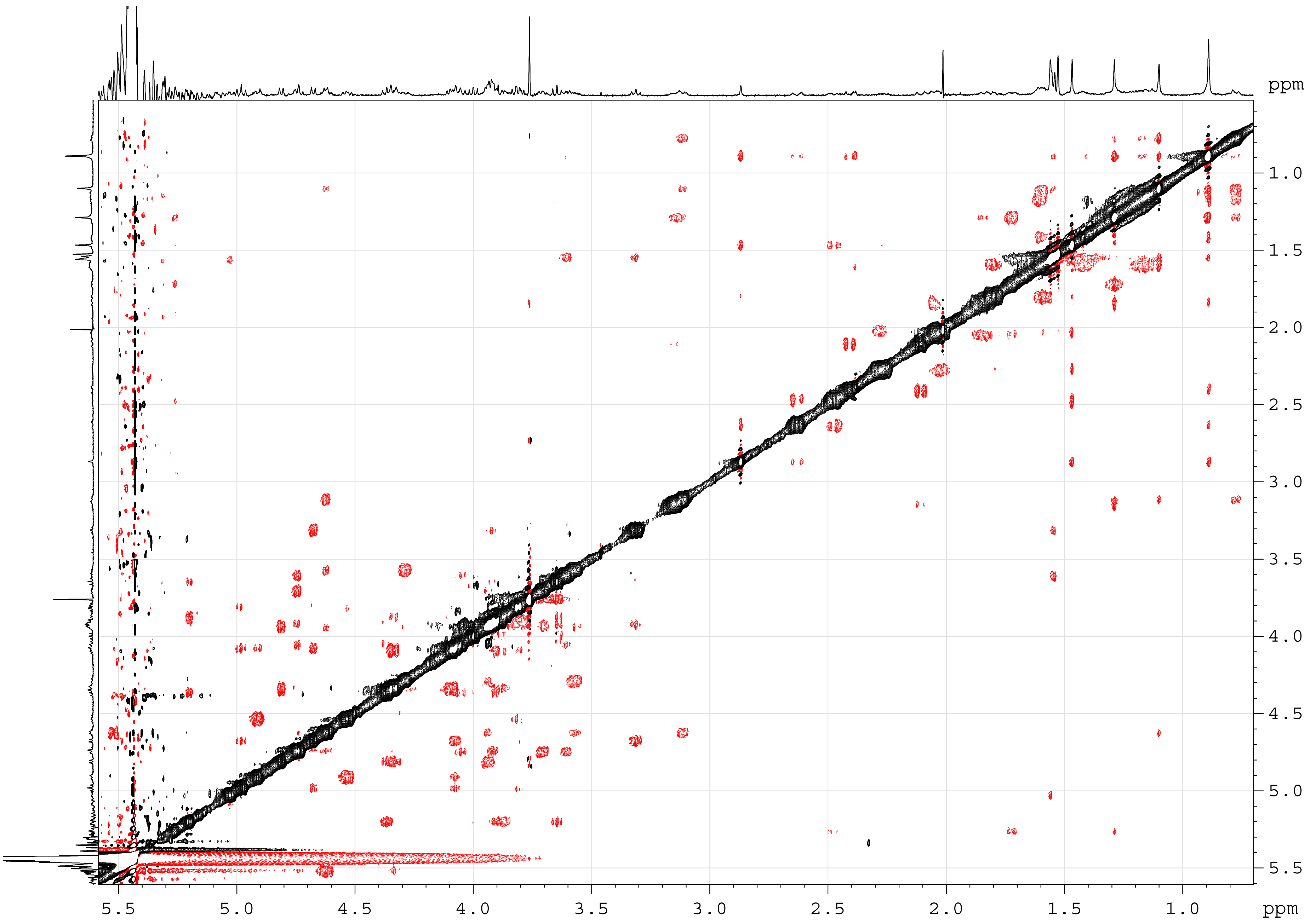

Figure S14. The ROESY (500.12 MHz) spectrum of chilensoside F (**2**) in C_5D_5N/D_2O (4/1)

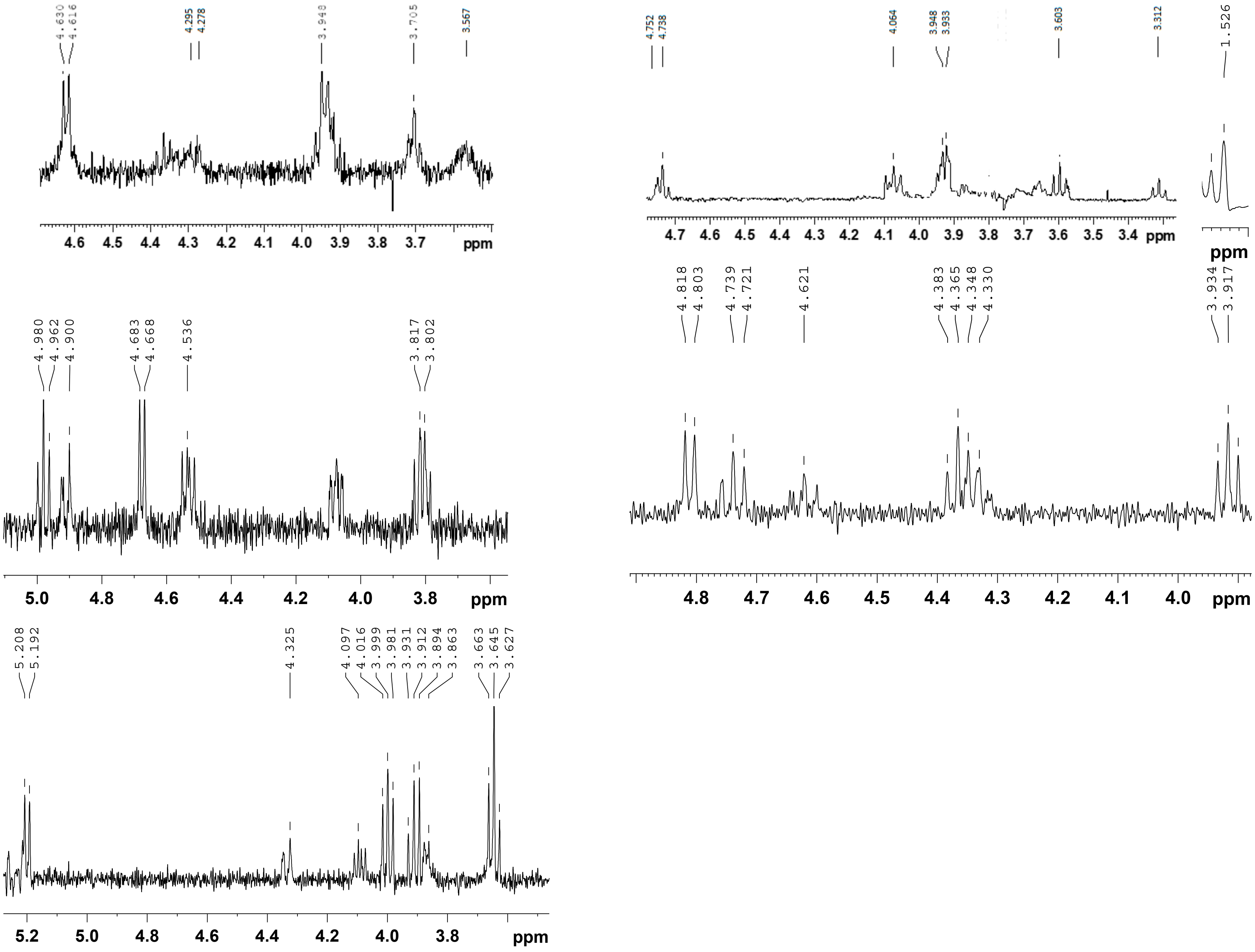

Figure S15. 1D TOCSY (500.12 MHz) spectra of Xyl1, Qui2, Glc3, Glc4, MeGlc5 of chilensoside F (**2**) in C_5D_5N/D_2O (4/1)

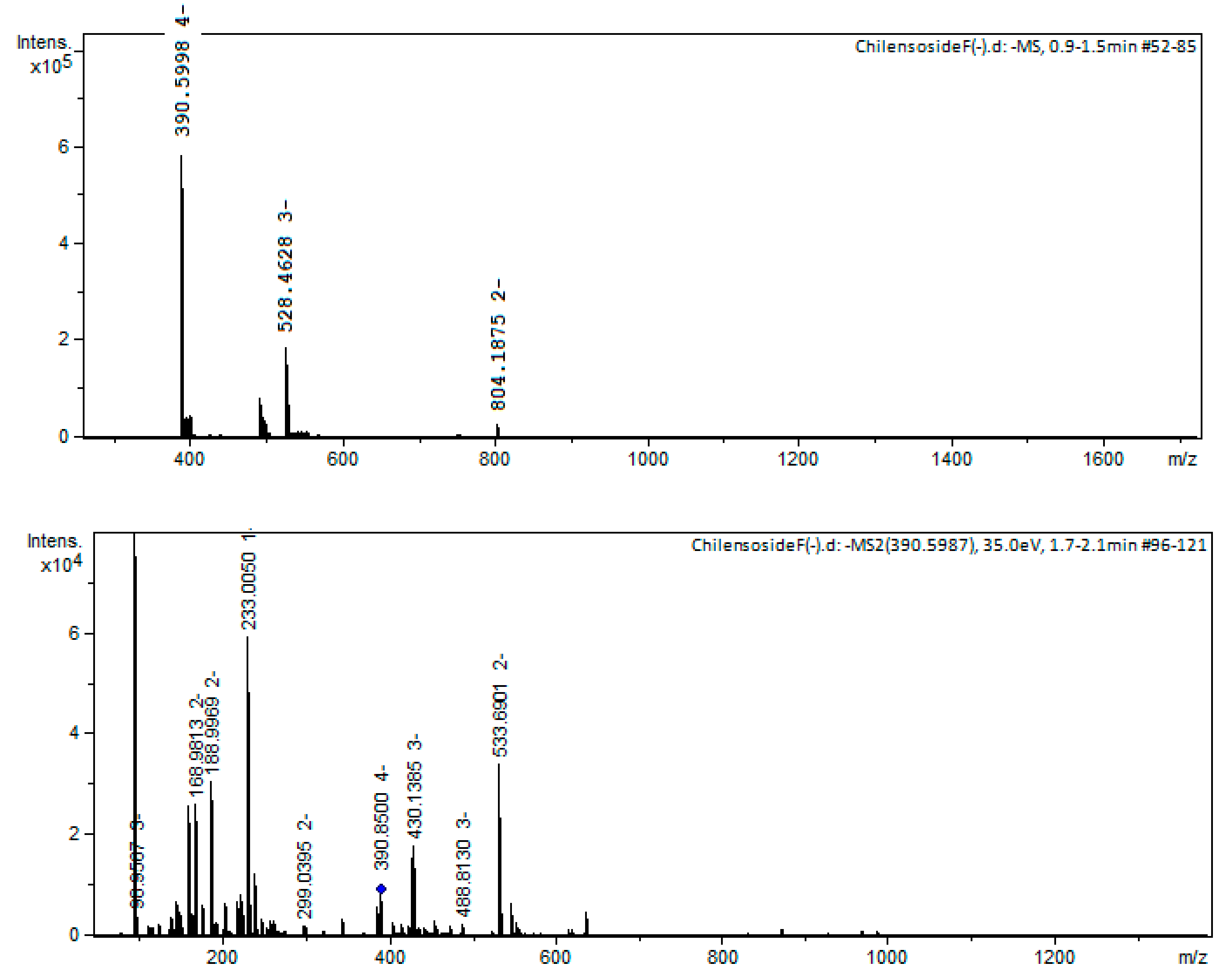

Figure S16. HR-ESI-MS and ESI-MS/MS spectra of chilensoside F (**2**)

Table S1. ^{13}C and ^{1}H NMR chemical shifts, HMBC and ROESY correlations of the aglycone moiety of chilensoside F (**2**).

Position	δ_C mult.[a]	δ_H mult. (*J* in Hz)[b]	HMBC	ROESY
1	36.0 CH_2	1.72 m		H-11
		1.29 m		H-3
2	26.7 CH_2	2.04 m		
		1.83 m		H-19, H-30
3	88.3 CH	3.12 dd (4.6; 11.8)		H-1, H-5, H-31, H1-Xyl1
4	39.4 C			
5	52.6 CH	0.77 brd (11.8)	C: 4, 19, 30	H-1, H-3, H-7
6	20.8 CH_2	1.59 m		
		1.41 m		H-8, H-30
7	28.2 CH_2	1.61 m		H-15
		1.13 m		H-5, H-32
8	38.6 CH	3.14 m		H-6
9	151.1 C			
10	39.5 C			
11	111.1 CH	5.28 brs	C: 10, 13	H-1
12	31.9 CH_2	2.63 brd (16.5)	C: 11, 18	H-17
		2.47 dd (5.9; 16.5)	C: 11, 14	
13	55.8 C			
14	42.0 C			
15	51.9 CH_2	2.40 d (16.0)	C: 13, 16, 17, 32	
		2.11 d (16.0)	C: 14, 16, 32	H-8
16	214.4 C			
17	61.2 CH	2.89 s	C: 12, 13, 16, 18, 20, 21	H-12, H-23, H-32
18	176.7 C			
19	21.9 CH_3	1.29 s	C: 1, 5, 9, 10	H-1, H-2, H-8, H-30
20	83.4 C			
21	26.6 CH_3	1.47 s	C: 17, 20, 22	H-12, H-17, H-23
22	38.6 CH_2	1.80 m		
		1.60 m		
23	22.9 CH_2	2.26 m		
		2.03 m		
24	123.7 CH	5.03 m		H-22
25	132.0 C			
26	25.5 CH_3	1.55 s	C: 24, 25, 27	H-24
27	17.4 CH_3	1.53 s	C: 24, 25, 26	H-23
30	16.3 CH_3	0.88 s	C: 3, 4, 5, 31	H-2, H-6, H-19, H-31
31	27.8 CH_3	1.10 s	C: 3, 4, 5, 30	H-3, H-5, H-6, H-30
32	20.5 CH_3	0.88 s	C: 8, 13, 14, 15	H-7, H-12, H-15, H-17

[a] Recorded at 176.04 MHz in C_5D_5N. [b] Recorded at 700.13 MHz in C_5D_5N.

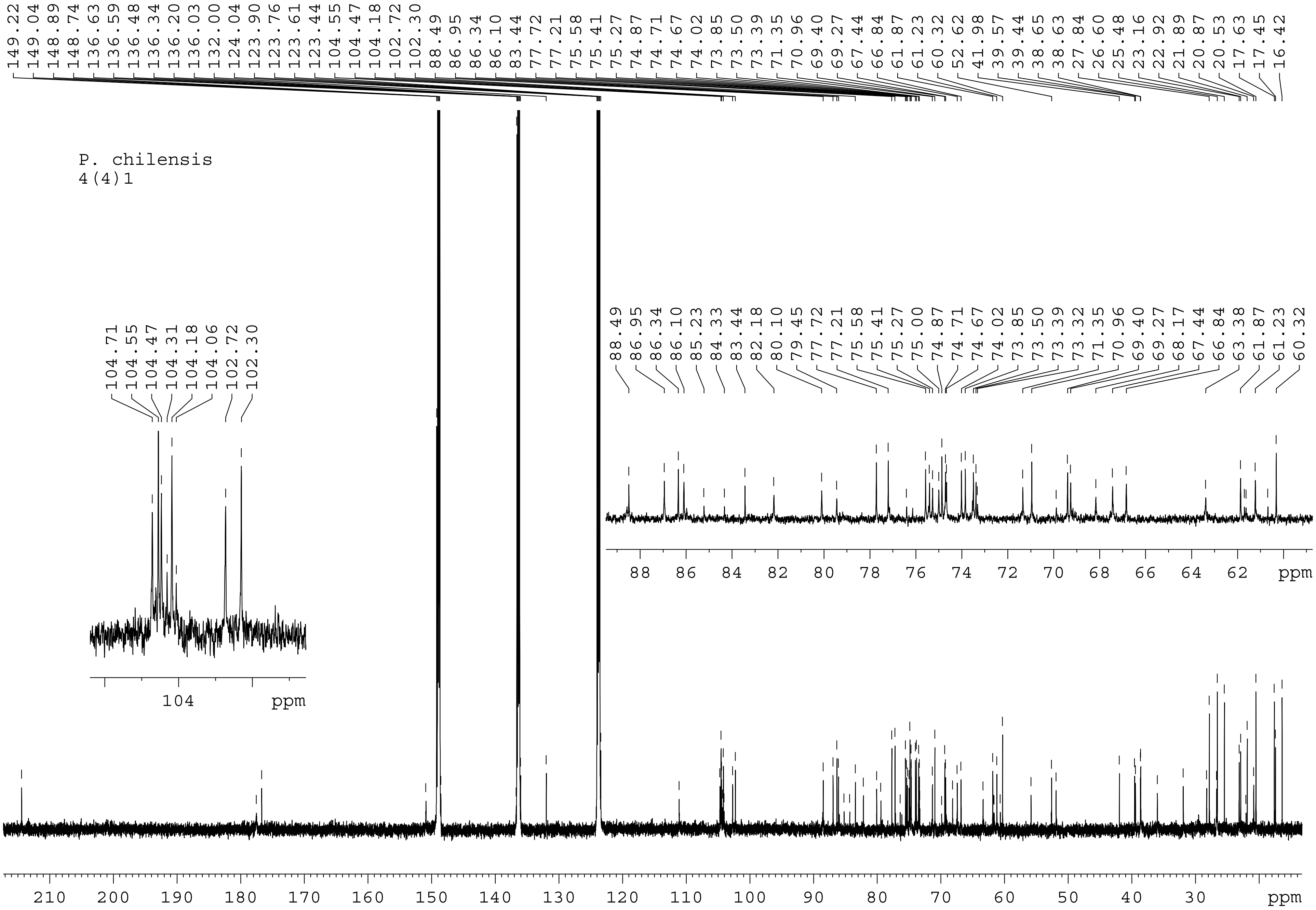

Figure S17. The ^{13}C NMR (125.67 MHz) spectrum of chilensoside G (**3**) in C_5D_5N/D_2O (4/1)

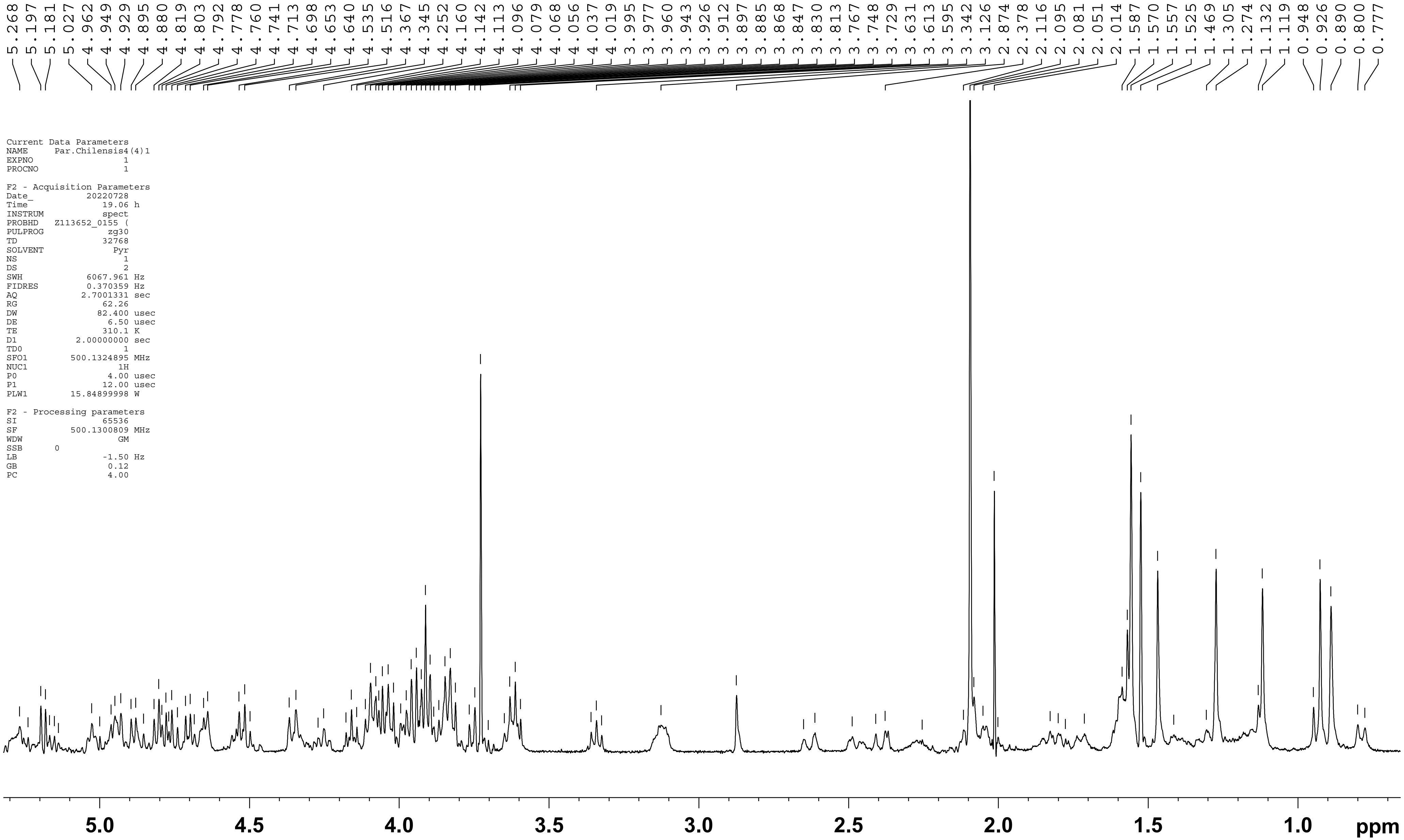

Figure S18. The ^{1}H NMR (500.12 MHz) spectrum of chilensoside G (**3**) in C_5D_5N/D_2O (4/1)

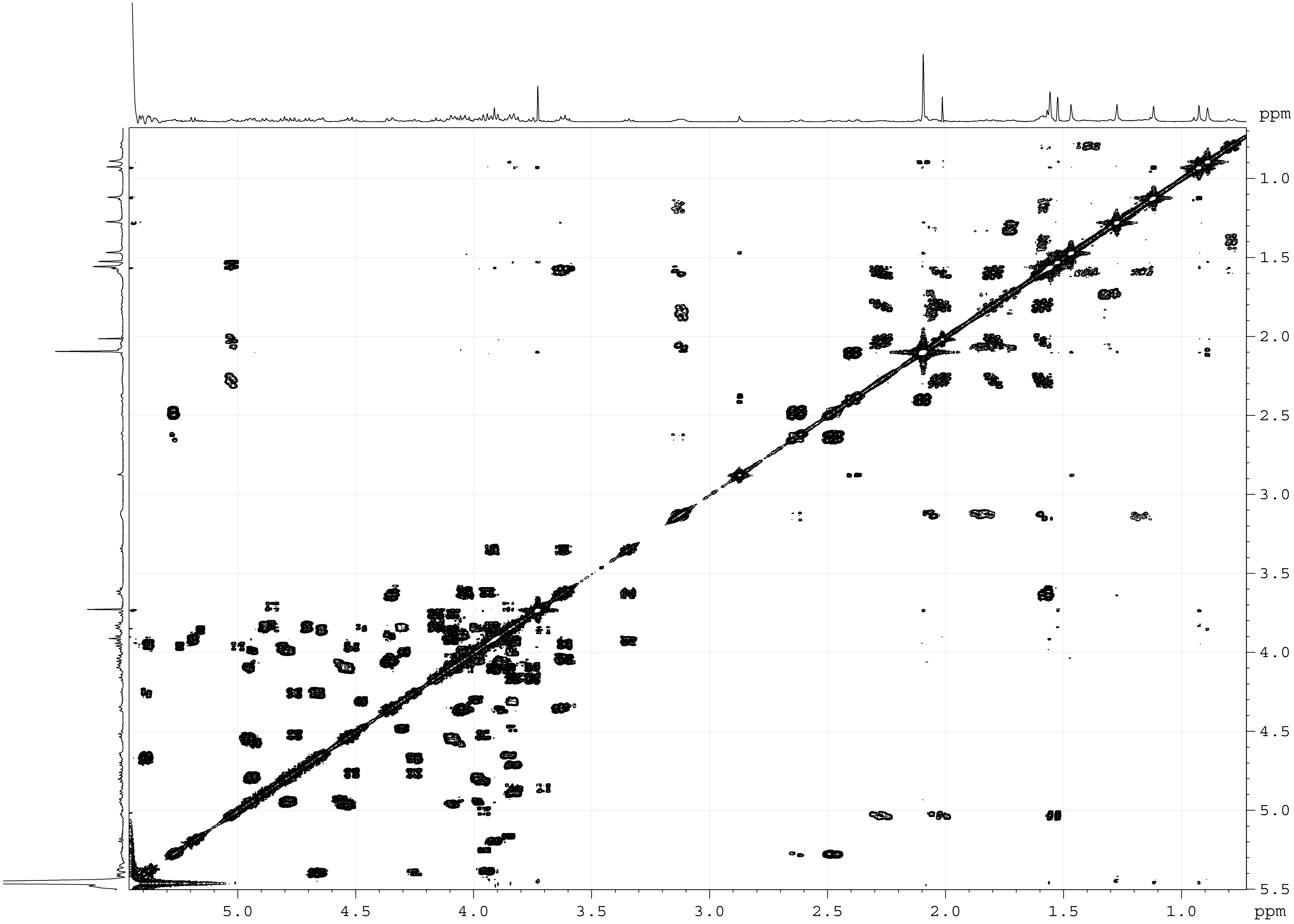

Figure S19. The COSY (500.12 MHz) spectrum of chilensoside G (**3**) in C_5D_5N/D_2O (4/1)

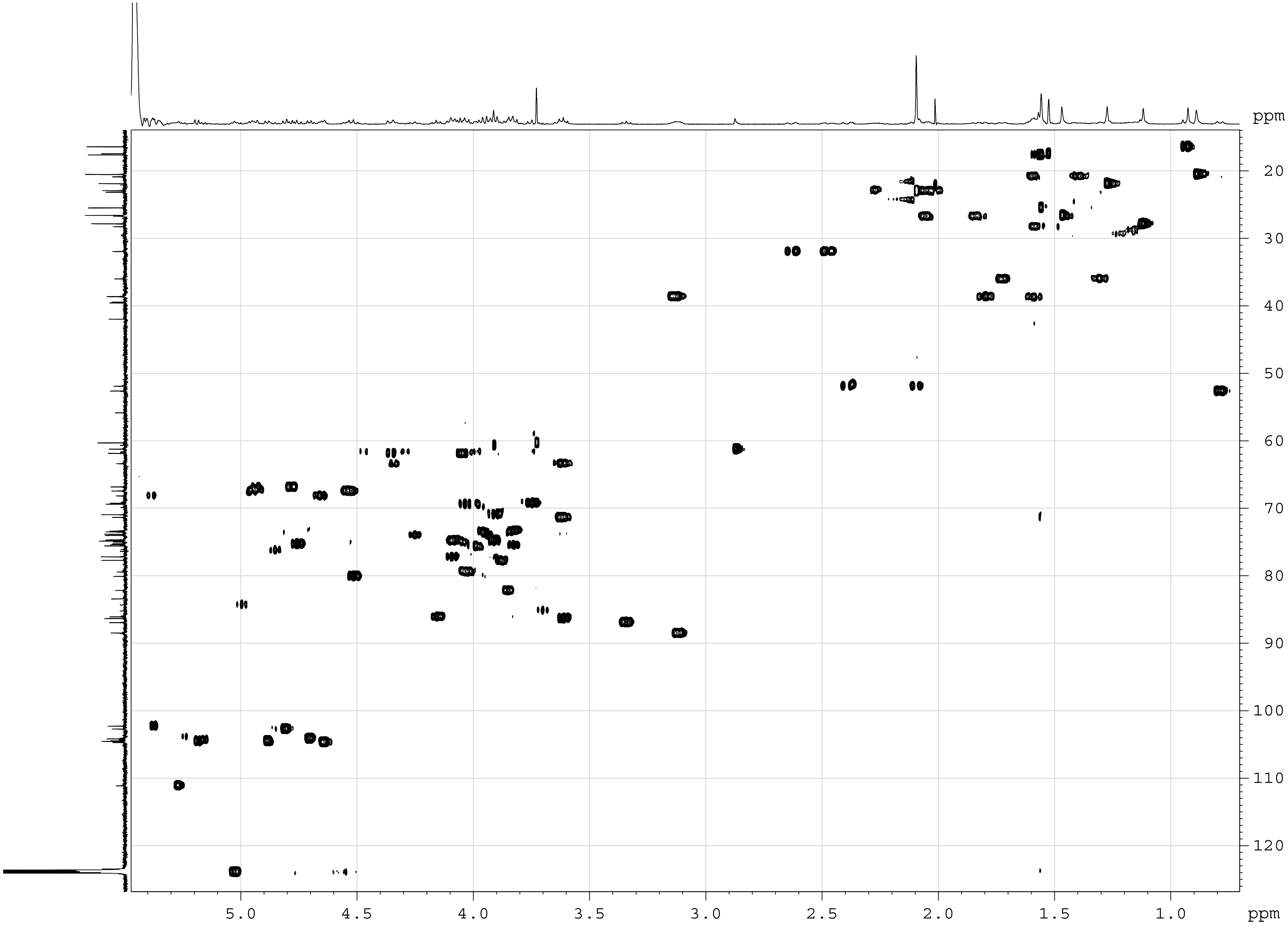

Figure S20. The HSQC (500.12 MHz) spectrum of chilensoside G (**3**) in C_5D_5N/D_2O (4/1)

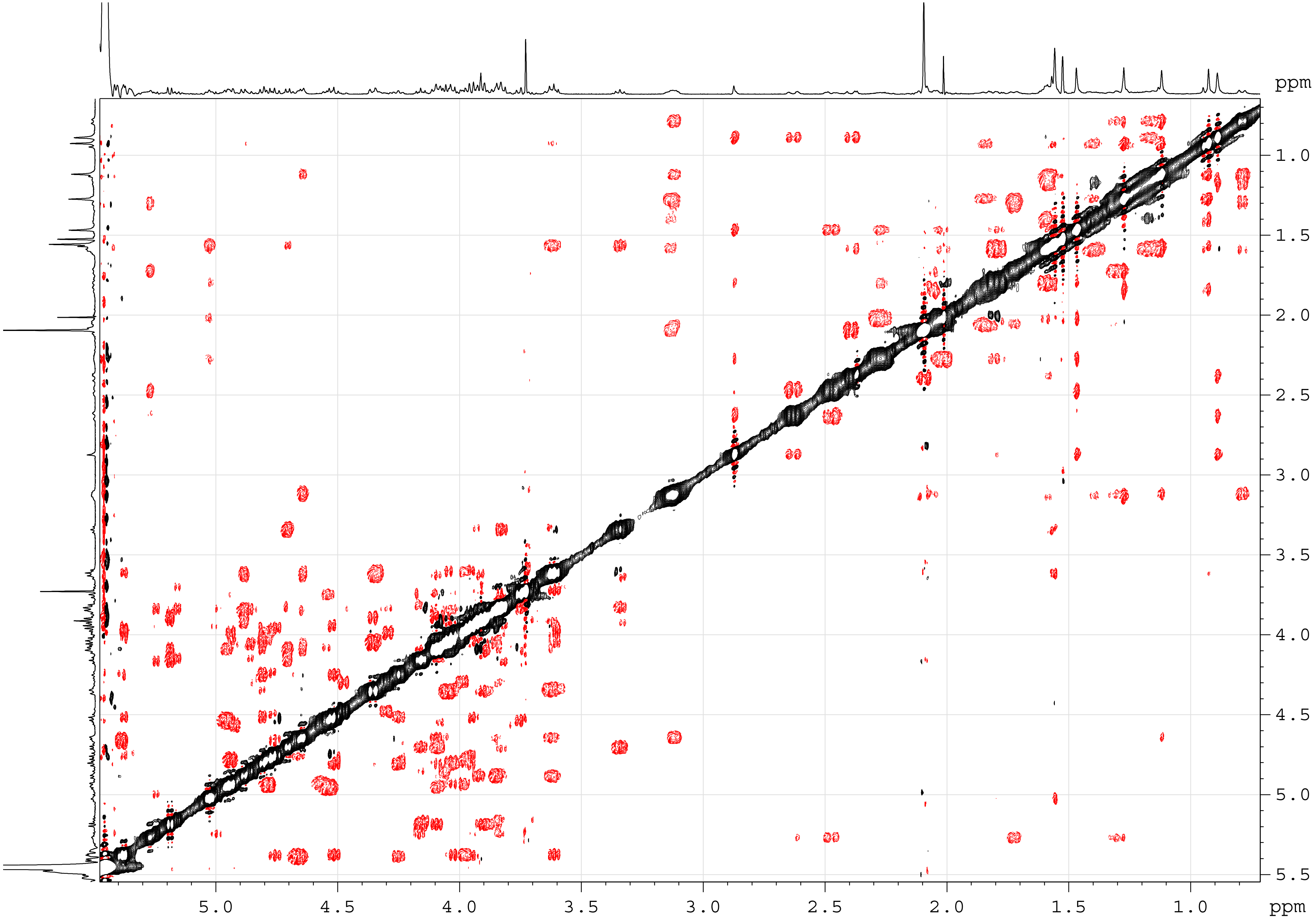

Figure S21. The ROESY (500.12 MHz) spectrum of chilensoside G (**3**) in C_5D_5N/D_2O (4/1)

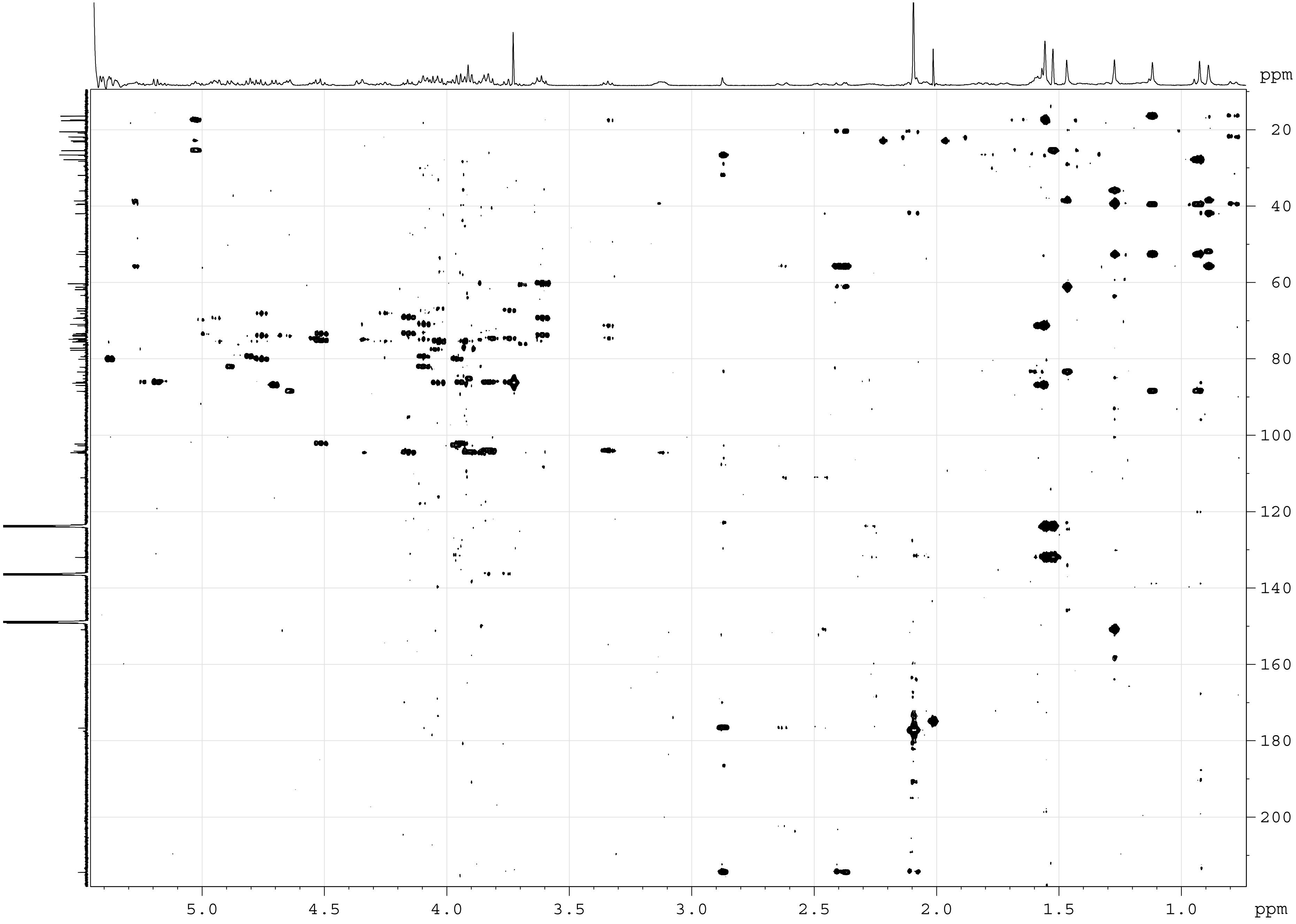

Figure S22. The HMBC (500.12 MHz) spectrum of chilensoside G (**3**) in C_5D_5N/D_2O (4/1)

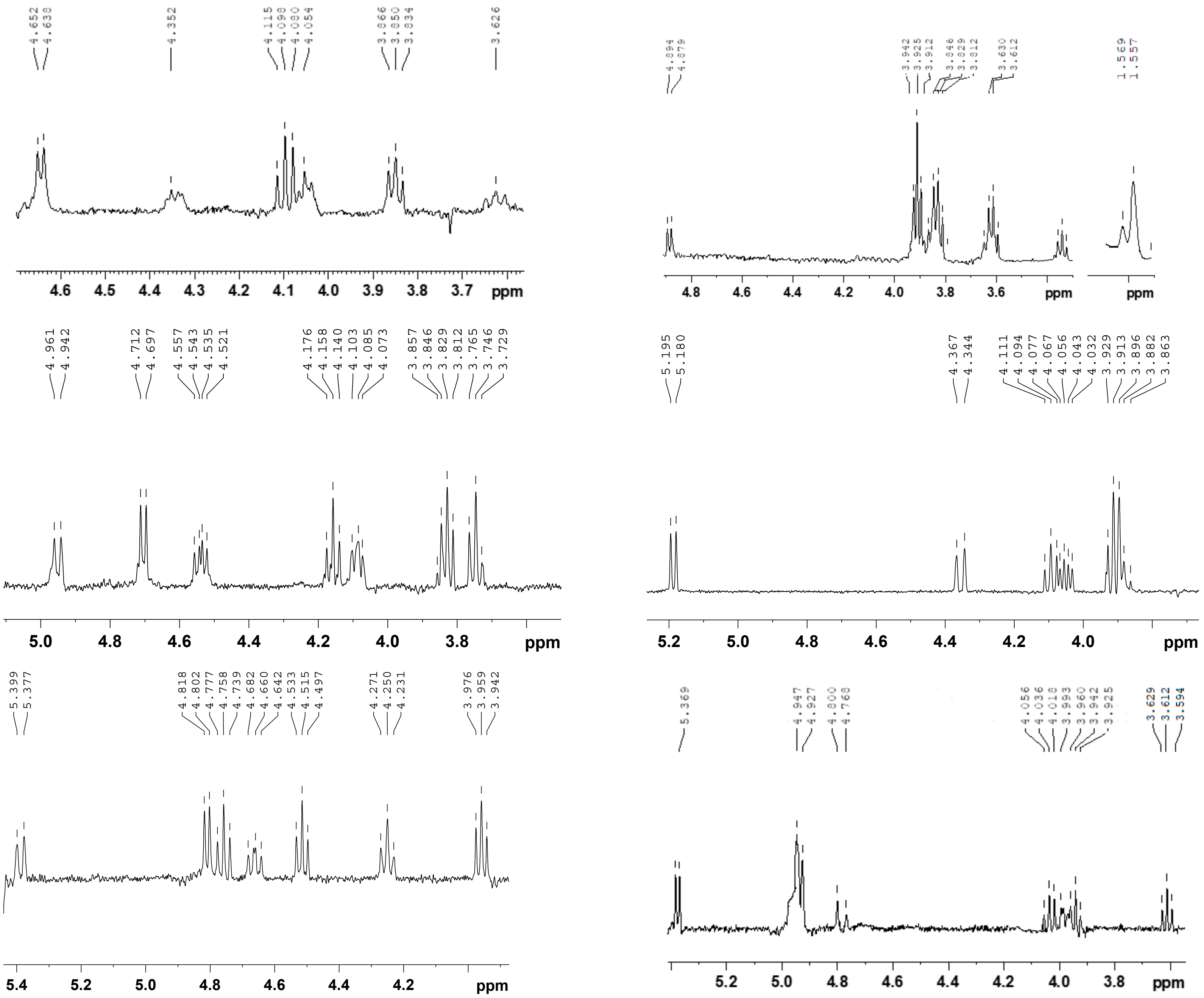

Figure S23. 1D TOCSY (500.12 MHz) spectra of Xyl1, Qui2, Glc3, Glc4, Glc5 and MeGlc6 of chilensoside G (**3**) in C_5D_5N/D_2O (4/1)

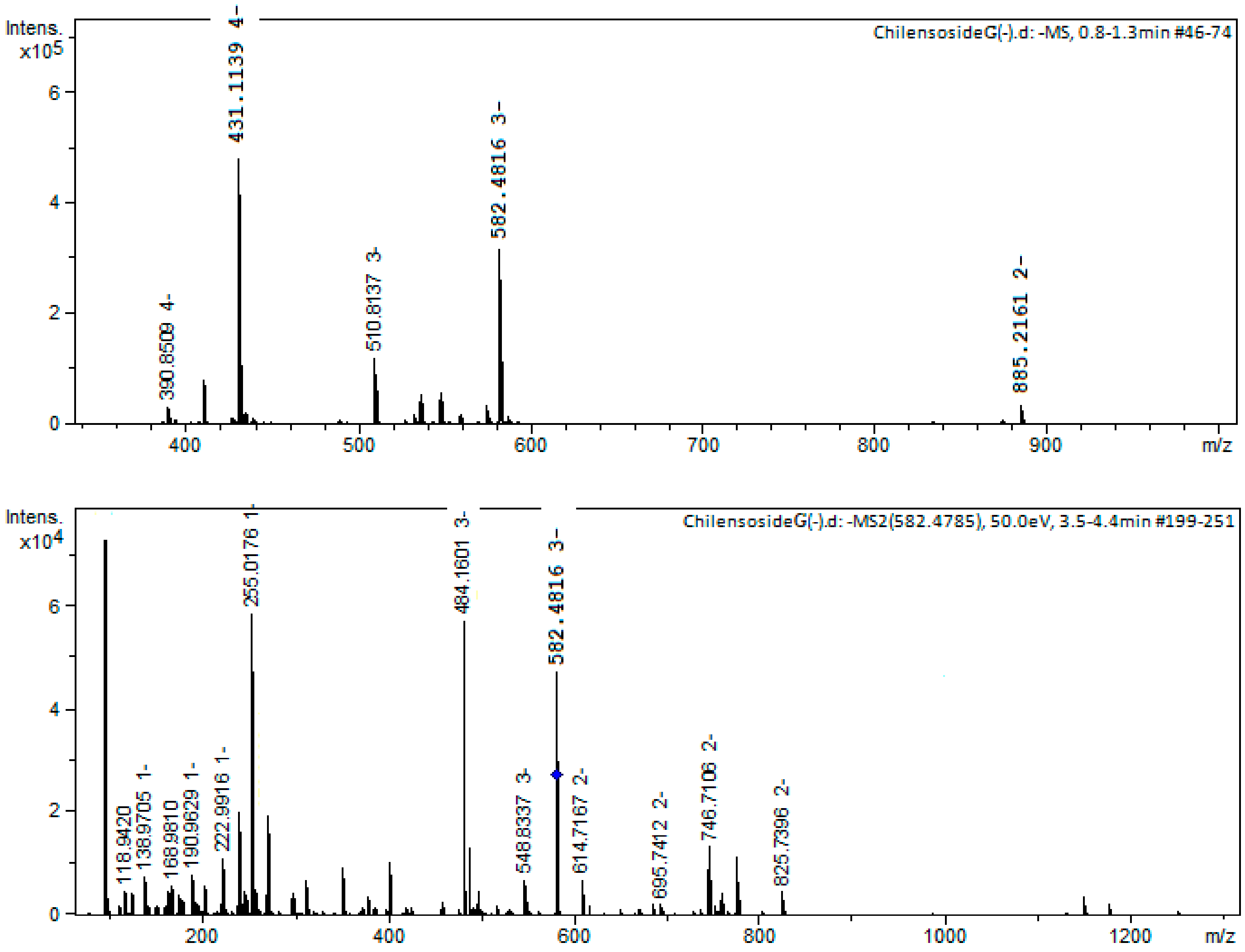

Figure S24. HR-ESI-MS and ESI-MS/MS spectra of chilensoside G (**3**)

Table S2. ^{13}C and ^{1}H NMR chemical shifts, HMBC and ROESY correlations of the aglycone moiety of chilensoside G (**3**).

Position	δ_C mult.[a]	δ_H mult. (*J* in Hz)[b]	HMBC	ROESY
1	36.0 CH_2	1.73 m		H-11
		1.32 m		H-3
2	26.7 CH_2	2.06 m		
		1.83 m		H-19, H-30
3	88.5 CH	3.11 dd (4.4; 11.5)		H-1, H-5, H-31, H1-Xyl1
4	39.4 C			
5	52.6 CH	0.78 brd (11.5)	C: 4, 6, 19, 30	H-1, H-3, H-7
6	20.9 CH_2	1.59 m		
		1.40 m		H-8, H-30
7	28.3 CH_2	1.60 m		H-15
		1.17 m		H-5, H-32
8	38.7 CH	3.13 m		H-6, H-15, H-19
9	151.1 C			
10	39.4 C			
11	111.3 CH	5.29 brd (5.2)	C: 10, 13	H-1
12	31.9 CH_2	2.65 brd (17.1)	C: 11, 18	H-17, H-32
		2.49 dd (6.0; 17.1)	C: 11, 14	H-17, H-21
13	55.8 C			
14	42.0 C			
15	51.9 CH_2	2.41 d (15.6)	C: 13, 16, 17, 32	
		2.10 d (15.6)	C: 14, 16, 32	H-8
16	214.4 C			
17	61.2 CH	2.90 s	C: 12, 13, 16, 18, 20, 21	H-12, H-23, H-32
18	176.8 C			
19	21.9 CH_3	1.29 s	C: 1, 5, 9, 10	H-1, H-2, H-8, H-30
20	83.4 C			
21	26.6 CH_3	1.48 s	C: 17, 20, 22	H-12, H-17, H-23
22	38.6 CH_2	1.80 m		
		1.60 m		
23	23.2 CH_2	2.29 m		H-21
		2.04 m		
24	124.0 CH	5.03 m		H-22
25	132.1 C			
26	25.5 CH_3	1.55 s	C: 24, 25, 27	H-24
27	17.4 CH_3	1.52 s	C: 24, 25, 26	H-23
30	16.4 CH_3	0.90 s	C: 3, 4, 5, 31	H-2, H-6, H-19, H-31
31	27.8 CH_3	1.10 s	C: 3, 4, 5, 30	H-3, H-5, H-6, H-30
32	20.5 CH_3	0.89 s	C: 8, 13, 14, 15	H-7, H-12, H-15, H-17

[a] Recorded at 125.67 MHz in C_5D_5N. [b] Recorded at 500.12 MHz in C_5D_5N.
